# Practical guidance for treatment of patients with diabetes using flash glucose monitoring: a pilot study

**DOI:** 10.1186/s13098-018-0364-z

**Published:** 2018-08-07

**Authors:** Keiko Koide, Koichiro Azuma, Jun Nojima, Kenichiro Kodama, Yoshihito Atsumi

**Affiliations:** 1 Diabetes Center, Eiju General Hospital, 2-23-16, Higashi-Ueno, Taito-ku, Tokyo, 110-8645 Japan; 2 Institute for Integrated Sports Medicine, Keio University School of Medicine, 35 Shinanomachi, Shinjuku-ku, Tokyo, 160-8582 Japan

**Keywords:** Accuracy, Certified diabetes educator, Flash glucose monitoring (FGM), Self-monitoring of blood glucose (SMBG)

## Abstract

**Background:**

Flash glucose monitoring (FGM) is a factory-calibrated, blood glucose measuring sensor system for patients with diabetes. We aimed to investigate the correlation between the sensor glucose (SG) value obtained using an FGM device and the traditional self-monitoring of blood glucose (SMBG) value.

**Methods:**

In 30 patients with diabetes under insulin treatment, SG and SMBG values were measured for 2 weeks, and the correlation between the values was analyzed.

**Results:**

The mean number of accumulated measurements of SG values was 1223.2 ± 193.0, whereas that of the SMBG values was 49.2 ± 21.3. Although SG and SMBG values showed a favorable correlation (*R*^*2*^ = 0.8413), SG values were lower than SMBG values by an average of 7.9 ± 29.8 mg/dL. The correlation patterns fell into four types: low type (SG values lower than SMBG values; *n* = 12), high type (SG values higher than SMBG values; *n* = 3), cross type (the slope of the two regression lines crossed at a certain measurement value; *n* = 14), and matching type (the values overlapped; *n* = 1).

**Conclusions:**

Recognition of the characteristic correlation patterns between SG and SMBG values is indispensable for certified diabetes educators to provide appropriate treatment guidance to patients with diabetes.

## Background

Patients with diabetes must maintain appropriate glycemic control while avoiding hypoglycemia in order to prevent microvascular complications and cardiovascular events [1, 2]. To attain appropriate glycemic control, continuous glucose monitoring (CGM) or flash glucose monitoring (FGM) is more useful than self-monitoring of blood glucose (SMBG), because an SMBG meter does not continuously measure blood glucose values [3, 4]. In a meta-analysis, patients with Type 1 diabetes mellitus using real-time CGM showed a greater reduction in hemoglobin A1c (HbA1c) values and exposure to hypoglycemia than those using an SMBG meter [5].

The FGM device is calibrated before shipment from the factory; it can provide blood glucose values to patients at any time while it is worn for 14 days. This means of interstitial glucose measurement has gained patient acceptance since it offers relief from fingertip glucose measurement. For Certified Diabetes Educators (CDEs), FGM is a potent educational tool, because by using it, we can visualize the effect of meals and exercise by confirming the time course change of blood glucose values that had not been visible until real-time GM such as FGM came into use.

Real-time GM device is, however, unable to measure blood glucose directly, and estimate it by measuring interstitial glucose level. Therefore, one issue to consider is the time lag between interstitial and blood glucose, and it has been reported to be around 5 min, which is clinically thought to be an acceptable indicator for blood glucose control [6]. However, some patients, who also measured their blood glucose values using an SMBG meter (SMBG values) while an FGM device was worn, complained that the values obtained using FGM (sensor glucose, SG values) were lower than SMBG values. On the contrary, a few SG values that could be falsely within the normoglycemic range occurred in patients with existing symptoms of hypoglycemia. Error grid analysis, which certifies the accuracy of the measured values, has been reported to show a high correlation between SG and SMBG values [7]. Nevertheless, when comparing the individual values of each patient, SG and SMBG values greatly deviated, even in the consensus error grid zones where both values had shown a favorable correlation in the aforementioned report. For each patient, the SG values obtained at the time of scanning greatly influence the patient’s everyday diabetes treatment; i.e., patients may misjudge how to treat themselves (for example, treating themselves as being hypoglycemic or increasing/decreasing the insulin dosage). Based on these reasons, we conducted the present pilot study using FGM and SMBG for 2 weeks in outpatients with diabetes who were receiving diabetes education using SMBG to analyze the specific correlation between SG and SMBG values.

## Methods

### Subjects

The subjects were 30 patients with diabetes (18 males, 12 females) under insulin treatment at our hospital; 17 subjects had type 1 diabetes mellitus, and 13 had type 2 diabetes mellitus, with a mean age and standard deviation of 55.3 ± 15.4 years. Mean body mass index was 23.9 ± 3.0 kg/m^2^, mean disease duration was 17.9 ± 12.6 years, and mean HbA1c value was 7.8 ± 0.8% (62 mmol/mol). Fifteen subjects suffered from retinopathy, and seven subjects had developed nephropathy. All participants were informed about the study and informed consent was obtained from all the participants. The Ethics Committee of Eiju General Hospital confirmed the morality and ethics of that study; No. 2015-16.

### Study design

The subjects wore a flash glucose monitoring device (FGM, FreeStyle Libre™ personal; Abbott Diabetes Care, Witney, UK) for 2 weeks, and conducted non-blinded measurement of their interstitial glucose levels (SG values). During the same period, the subjects also conducted measurement of capillary blood glucose values (SMBG values) using an SMBG meter GLUCOCARD G Black™ (ARKRAY Inc., Kyoto, Japan). SMBG was mostly performed before meals to avoid rapid rises or falls in BG. The number of accumulated measurements of SG and SMBG values in individual patients was counted, and the mean number of measurements was calculated.

### Data analysis

SG values obtained by the reader of an FGM device and SMBG values obtained by a SMBG meter were exported to an original data management software, MEQNET SMBG Viewer (SV), which was developed under collaborative work by ARKRAY and the Diabetes Center of Eiju General Hospital, Tokyo, Japan, and will be released soon from the ARKRAY website, http://www.arkray.co.jp/japanese/products/support/soft/smbg_viewer_ver2.html.

The SV combines the data from both devices, according to the time of day, and displays graphs of daily fluctuations of both SG and SMBG values on the same screen, and simultaneously provides a comparative indication of descriptive statistics [minimum, maximum, mean and median values, hypoglycemia rate, hyperglycemia rate, SD and interquartile range (IQR)]. Furthermore, the SV has a function to graphically display the correlation between SG and SMBG values. Since SG values were saved only every 15 min, the SV software could not exactly match the timing of SG values with that of SMBG value measurements. Therefore, considering that the time lag of glucose from intravascular to interstitial compartment has been reported to be around 5–6 min [6], the SV software chose the proximate subsequent SG values to SMBG value measurements. Key characteristics of FGM for health care providers are that the visual graphs and calculated results can be printed out and subsequently effectively used for patient guidance in relation to lifestyle improvement or SMBG measurement timing and for other purposes. In this study, we examined the correlation between SMBG values and the corresponding SG values. SMBG values of less than 40 mg/dL and those of 500 mg/dL or more were excluded from the comparison.

The correlation patterns of individual SG and SMBG values displayed on the SV were categorized by comparison between the diagonal line and the slope of the regression line. Analysis was also conducted on the difference in SG and SMBG values, using factors such as type of diabetes, degree of SG value fluctuations (indicated with arrows), number of days elapsed after the start of wearing an FGM device, and low/high ranges of SG/SMBG values.

### Statistical methods

For statistical analysis, two-sample t-test was used, and a level of significance of less than 5% was established using a two-sided test.

The mean absolute relative difference (MARD) is to see the difference in individual values of each patient between SG and SMBG values and was defined as

$${\text{MARD }}\left( \% \right)\, = \, 100\, \times \,|{\text{SG value}}{-}{\text{SMBG value}}|/{\text{SMBG value}}$$

## Results

In 30 subjects who wore an FGM device, the mean number of accumulated measurements of SG values was 1223.2 ± 193.0, whereas that of the SMBG values was 49.2 ± 21.3. For SG value recording, the mean number of daily scans was 11.8 ± 9.8.

**Fig. 1:**
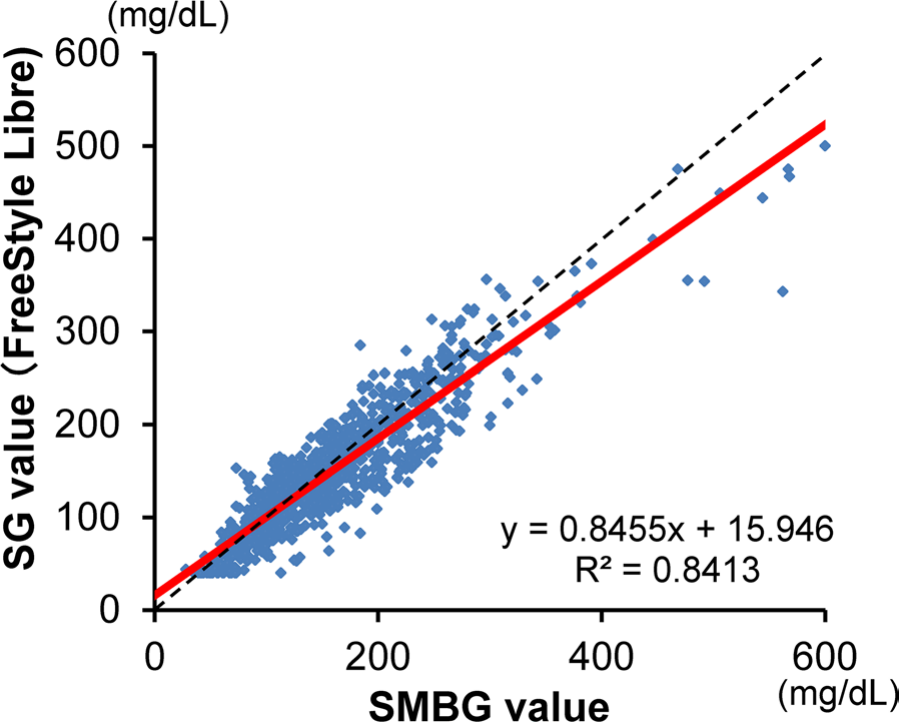
Correlation between SG and SMBG values in all subjects (*n* = 30). SG values and corresponding SMBG values showed a favorable correlation (R^2^ = 0.8413). SG values were lower than SMBG values by an average of − 7.9 ± 29.8 mg/dL (− 3.5 ± 19.0%). *SG* sensor glucose, *SMBG* self-monitoring of blood glucose

SG values and the corresponding SMBG values showed a favorable correlation (R^2^ = 0.8413) (Fig. 1). Compared to SMBG values, SG values were lower by an average of − 7.9 ± 29.8 mg/dL (− 3.5 ± 19.0%). However, on an individual level, SG and SMBG values showed a wide range of deviation of − 219 to + 101 mg/dL (− 64.6 to + 109.6%), and MARD was 15.3 ± 6.7% (Type 1 diabetes: 15.0 ± 4.5%, Type 2 diabetes: 15.7 ± 8.5%).

**Fig. 2:**
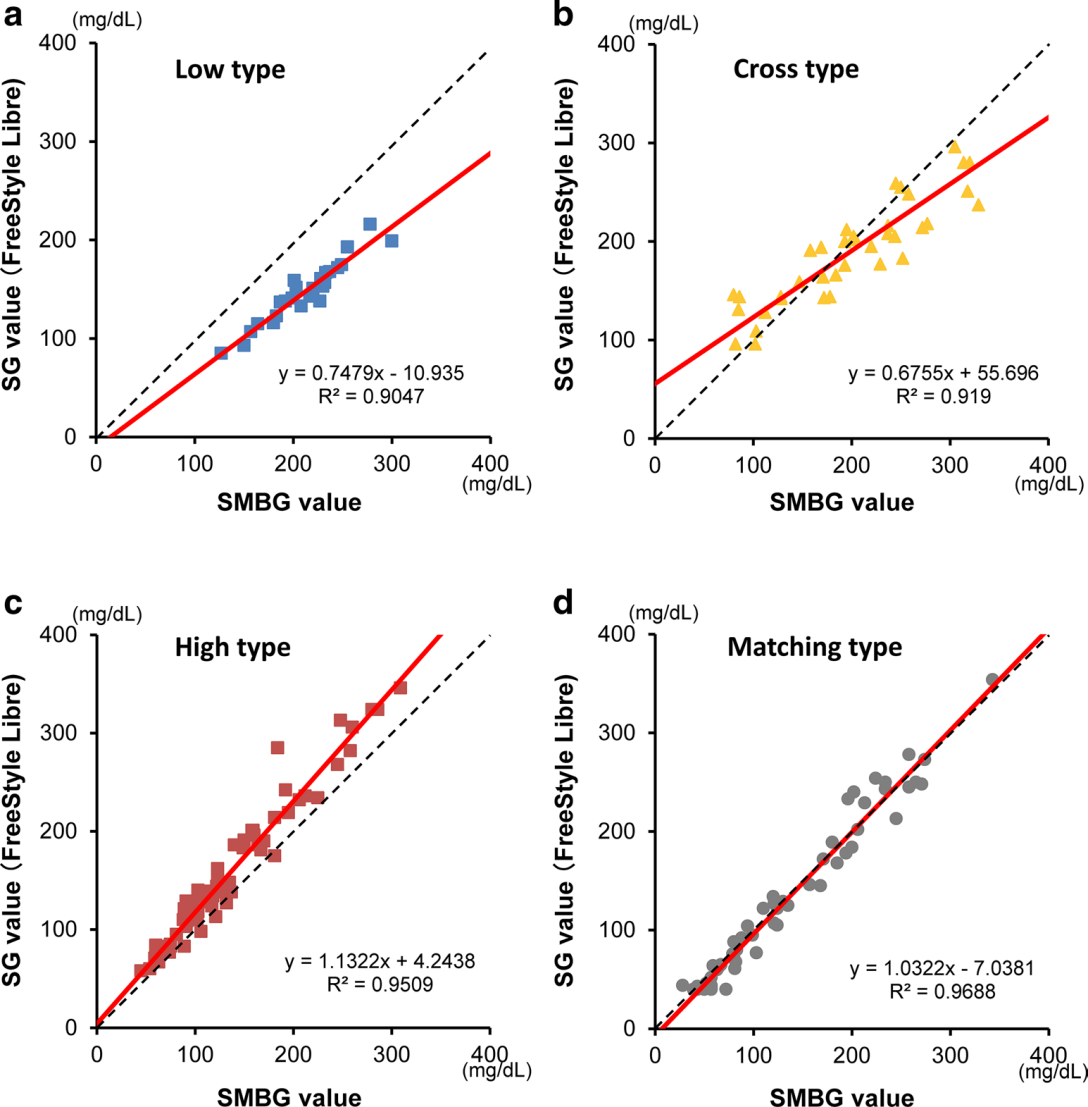
The four types of regression lines observed are each illustrated by a representative case. **a** (Top left) Low type, when SG values were lower than SMBG values (*n* = 12). **b** (Top right) Cross type, when the slope of the two regression lines crossed at a certain measurement value (*n* = 14). **c** (Bottom left) High type, when SG values were higher than SMBG values (*n* = 3). **d** (Bottom right) Matching type, when the values overlapped (*n* = 1). *SG* sensor glucose, *SMBG* self-monitoring of blood glucose

The correlation patterns spontaneously fell into four types: low type, when the regression line was lower than the diagonal line, in other words, when SG values were lower than SMBG values (*n* = 12); cross type, when the lines crossed at a certain measurement value (*n *= 14); high type, when the regression line was higher than the diagonal line (*n* = 3); and matching type, when the values overlapped (*n* = 1) (Fig. 2a–d). In the cross type, all subjects showed low SG values only when SMBG values were high. There were no differences in baseline patient characteristics, such as age, body mass index, duration of diabetes, HbA1c value, and microvascular complications, between the cross type and low type. Moreover, four patients, who had a chance to wear another FGM device and thereby repeated the same data analysis, showed correlation patterns identical to those observed in the first analysis.

**Fig. 3:**
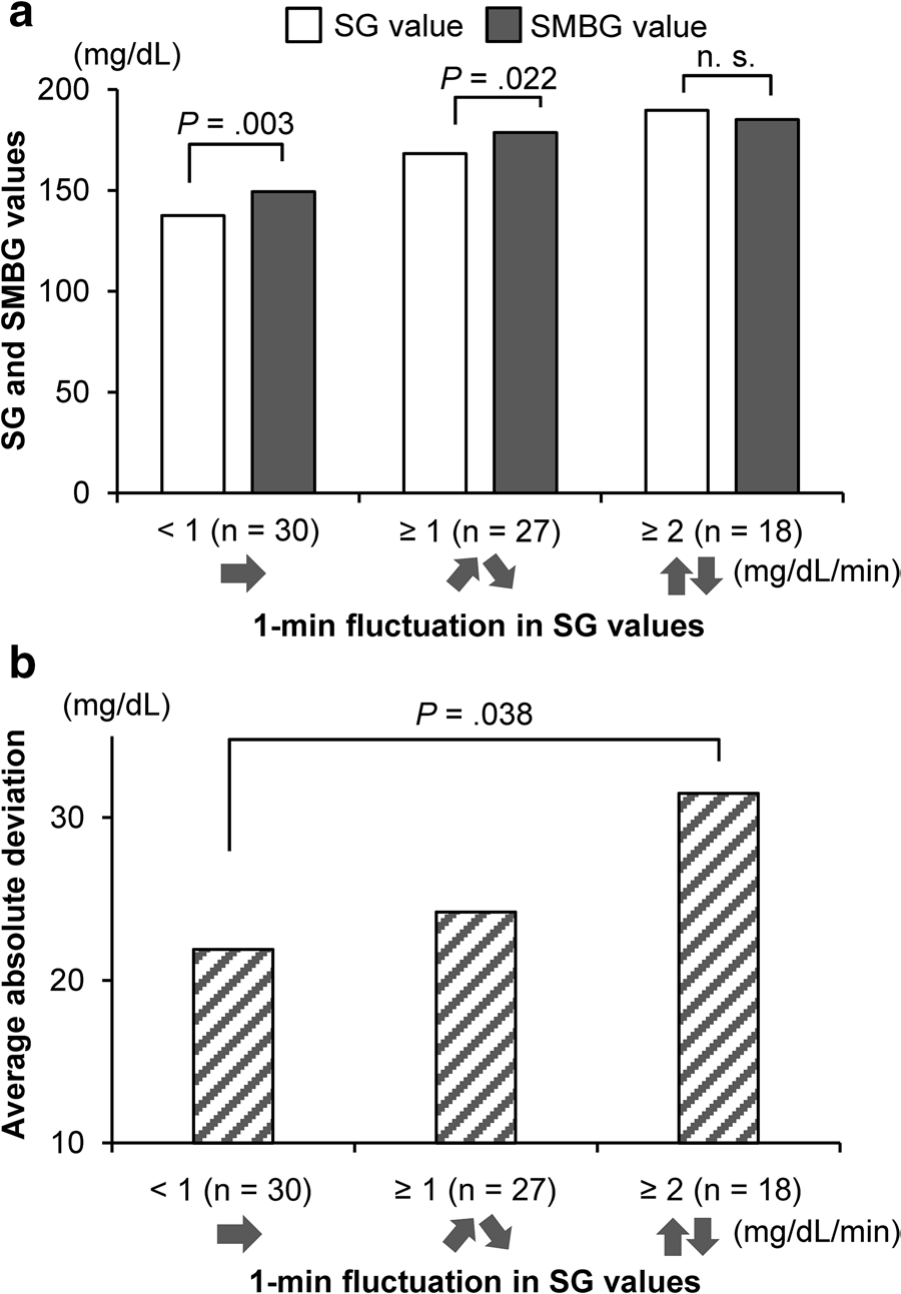
Comparison of deviation between SG and SMBG values by degree of SG fluctuations. **a** A significant difference between SG and SMBG values was found when SG fluctuation was < 1 mg/dL and ≥ 1 mg/dL, whereas no significant difference was found when SG fluctuation was ≥ 2 mg/dL/min. **b** A large average absolute deviation was found with SG fluctuation ≥ 2 mg/dL with a significant difference compared to SG fluctuation < 1 mg/dL (*p *= 0.038). *SG* sensor glucose, *SMBG* self-monitoring of blood glucose

In order to examine the relationship between the degree of SG value fluctuation and the deviations between SG and SMBG values, the difference between SG and SMBG values was compared based on the fluctuation patterns indicated by FGM. These patterns included a horizontal arrow (→) when the 1-min fluctuation in SG value was < 1 mg/dL, a diagonal arrow going up or down (↗↘) when the fluctuation was ≥ 1 mg/dL, and an arrow going up or down (↑↓) when the fluctuation was ≥ 2 mg/dL. The results showed that a significant difference between SG and SMBG values was found when SG fluctuation was < 1 mg/dL/min and ≥ 1 mg/dL/min, whereas no significant difference was found when SG fluctuation was ≥ 2 mg/dL/min (Fig. 3a). Nevertheless, in view of the average absolute deviation between SG and SMBG values, a large deviation was found with SG fluctuation ≥ 2 mg/dL/min compared to SG fluctuation < 1 mg/dL/min (*p* = 0.038) (Fig. 3b).

**Fig. 4:**
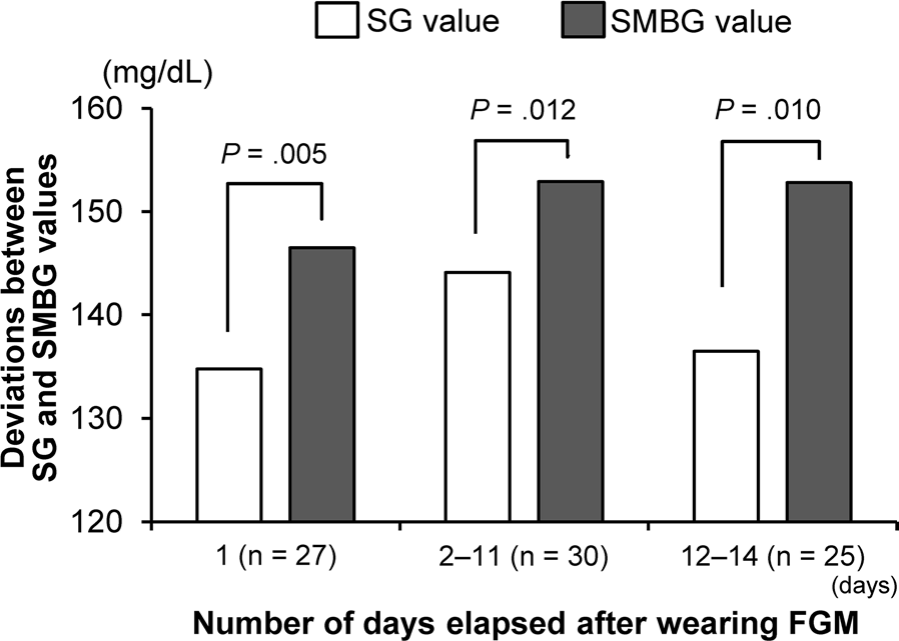
Comparison of the number of days which elapsed while wearing FGM. Comparisons of SG and SMBG values on Day 1, Days 2–11 and Days 12–14 all showed significantly lower SG values. The widest range of deviation was observed on Day 1. *FGM* flash glucose monitoring, *SG* sensor glucose, *SMBG* self-monitoring of blood glucose

To examine the relationship between the number of days from the start of wearing the FGM device and the deviation between SG and SMBG values, we compared the differences between SG and SMBG values on Day 1, Days 2–11 and Days 12–14 after the start of wearing the FGM device. During all of the periods, the SG values were significantly lower than SMBG values. Among the three periods, the SG value on Day 1 showed the most significantly lower readings (*p* = 0.005) (Fig. 4).

**Fig. 5:**
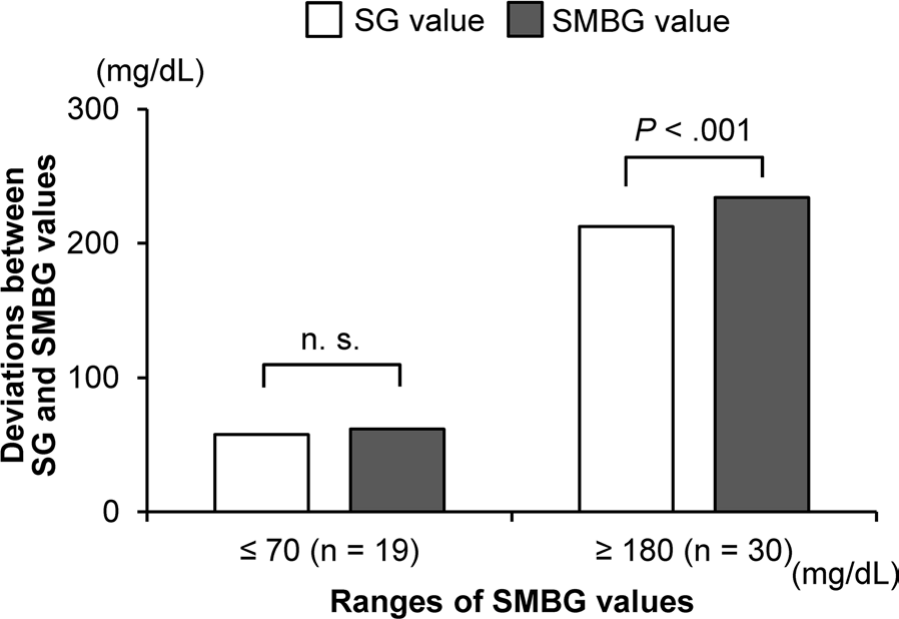
Comparison of low/high ranges of SG/SMBG values. The comparison between ranges of SG and SMBG values ≤ 70 mg/dL and ≥ 180 mg/dL revealed a wider range of deviation in the range ≥ 180 mg/dL. *SG* sensor glucose, *SMBG* self-monitoring of blood glucose

We also examined the relationship between low/high blood glucose values and SG/SMBG values, concerning the difference between SG and SMBG values at the time when SMBG values were ≤ 70 mg/dL or ≥ 180 mg/dL. SG values were significantly lower than SMBG values in the range ≥ 180 mg/dL (*p* = 0.001) (Fig. 5). However, despite the deviation between SG and SMBG values, when comparing the absolute values of difference against the mean SMBG values by percentage, no significant difference was found in both blood glucose ranges of ≤ 70 mg/dL and ≥ 180 mg/dL (≤ 70 mg/dL: 6.7%, ≥ 180 mg/dL: 9.3%).

When comparing the correlation between SG and SMBG values in patients with Type 1 and Type 2 diabetes, both types demonstrated the cross type, but the variance was larger in Type 1 diabetes. However, when excluding the values of a patient with Type 1 diabetes, who underwent total pancreatectomy, and was receiving prednisolone by intravenous drip infusion due to pancreatic cancer, the variance was reduced. In this patient, abnormally high values were observed in the blood glucose fluctuation indices, with markedly high risk values for hyperglycemia (average daily risk range: 56.4, low blood glucose index: 3.6, high blood glucose index: 21.4, IQR: 148, SD: 102.6) (data not shown).

## Discussion

Although a high overall correlation was observed between the SG and SMBG values in our study, both matching and deviating values were observed when comparing individual SG and SMBG values. These deviations were found to be related to various factors, such as the diabetes type, degree of blood glucose fluctuation, number of days after the start of wearing the FGM device and low/high blood glucose ranges of SG/SMBG values.

A large difference between SG and SMBG values on Day 1 of wearing the FGM device has been reported [8]. Factors, such as the following can be considered: SG values indicate interstitial glucose values, the precision of the algorithm displayed as blood glucose values, the measurement time-lag between SG and SMBG values [9, 10], the mounting position of FGM device and the margin of error of the SMBG meter. Our results also suggested that the range of deviation becomes larger in certain patients with labile diabetes who experience extreme blood glucose fluctuations, and in those with abnormally high HbA1c values of over 10% (> 86 mmol/mol). It is reported that FGM can replace SMBG because the comparison of values obtained using FGM and SMBG revealed no difference in the change of HbA1c values in the two groups, and that the FGM group experienced fewer hypoglycemic events [11–13]. However, FGM must be used with utmost care since deviations do occur in individual measurement values.

In the present study, we were able to categorize the subjects into four types of correlation patterns (low type, cross type, high type, and matching type) based on their SG and SMBG values. The reason why a constant correlation tendency between SG and SMBG values was not observed cannot be attributed to the existence of a single cause for the deviations. Since patients with the low type (*n* = 12, Fig. 2a) comprised 40% and those with the high type (*n* = 3, Fig. 2c) comprised 10%, it is likely that SG values obtained using FGM show lower values than those obtained by conventional SMBG measurement. In the future, we plan to recruit a suitable number of patients for analysis, categorize the patients into these four types, and then investigate whether or not any specific tendency which determines the correlation pattern can be revealed.

FGM has been demonstrated to be an effective means of analysis of glucose exposure, variability and hypoglycemia risk [14–16]. It is an innovative device because it can record real time measurement values throughout the period it is worn (for 2 weeks) without any pain, and patients do not have to perform fingertip blood glucose measurements, as calibration of the device is completed before factory shipment [8, 17]. Improvement in the QOL of patients with diabetes is expected since patients are relieved of the pain of fingertip blood glucose measurements [18, 19]. Despite the belief that FGM serves as a good solution for patients receiving multiple daily insulin therapy, our results suggest that fingertip blood glucose measurement should be performed in addition to FGM measurement.

In summary, it is necessary to understand the characteristics of SG values obtained in the present study, and to provide treatment guidance to patients with diabetes for continuous treatment using FGM, while also conducting SMBG measurements in a complementary manner. The following are assumed to be situations in which FGM and SMBG measurements complement each other, and thus, CDEs should pay attention to the following points when providing guidance to patients [20].

- For patients with the low type of correlation pattern, if SG values are in the low range or ≤ 70 mg/dL with a downward arrow, instruct them to measure SMBG values without excessive treatment for hypoglycemia unless they have symptoms of hypoglycemia. Even when SG values are within the normal range, advise them to continue conducting fingertip blood glucose measurements without reducing the level of self-management of meals and exercise.
- For patients with the cross type correlation pattern, instruct them not to overlook hypoglycemia at points below the crossing, and not to reduce the level of self-management at points above the crossing, by advising them to check fingertip blood glucose values at times when hyperglycemia may be likely to occur, such as after meals.
- For patients with the high type of correlation pattern, instruct them to pay careful attention to possible hypoglycemia symptoms, even if SG values are within the normal range. If SG values are ≥ 180 mg/dL with an upward arrow, instruct them not to reduce the meal amount or increase the insulin dosage, rather advise them to take the fingertip blood glucose value measurement.

Although both CGM and FGM are possible replacements for SMBG, it is important to instruct patients to appropriately conduct at least the minimum number of fingertip blood glucose measurements which is necessary from the standpoint of patient safety [21]. The accuracy of blood glucose values obtained by CGM or FGM is currently considered acceptable if the values fall within the ranges of A and B shown by error grid analysis [7]. However, given the fact that when using these devices in clinical practice, the individual SG and SMBG values deviate at certain measurement points, the safe use of CGM or FGM must be attained through knowledge of the correlation patterns revealed in the present study. Thus, explicit instructions given by a CDE on the proper use of innovative FGM devices are necessary for patients with diabetes.

In order to identify the correlation pattern between SG and SMBG values, fingertip blood glucose measurements should be recommended while wearing the FGM device. If a patient continues to exhibit the same correlation pattern, fingertip blood glucose measurement would be unnecessary for subsequent identification of the correlation pattern. However, since an FGM device was worn only one time in the current study, this issue remains unclear.

A limitation of this study is that this had a cross sectional design and we were thus unable to carry out a long-term observation to clarify whether or not the correlation patterns might be attributable to personal characteristics or differences in sensor lots. Though only 4 patients took repeated analyses and the number of patients was too small, identical correlation patterns were observed within the same subjects with repeated analyses employing different lots of FGM devices. Therefore, we consider it to be more likely that unknown characteristics of the patients, such as subcutaneous tissue conditions, rather than the FGM devices themselves, might have affected the observed glucose-sensing patterns. If patient-specific patterns can be identified, more safe and effective usage of FGM could be established. The greatest advantage of using an FGM is that patients can understand the glucose trend, which directly leads to appropriate treatment and consistent everyday living. Specifically, a CDE should instruct patients with asymptomatic hypoglycemia or large blood glucose fluctuations regarding the correlation patterns indicated in the present study, and then help them to utilize the patterns. Further review including more patients with analysis on a long-term basis is needed.

## Conclusions

Despite an overall favorable correlation between SG values obtained using FGM and SMBG values obtained using SMBG, the comparison among individual measurement values revealed deviations, which could be categorized into four patterns. It is necessary for CDEs to provide guidance to patients with diabetes, including the recommended timing of SMBG measurements, through confirmation of the specific characteristics of SG values obtained using FGM and observation of the changes in sensor glucose values.

## Abbreviations

FGM: flash glucose monitoring
SG: sensor glucose
SMBG: self-monitoring of blood glucose
CGM: continuous glucose monitoring (CGM)
HbA1c: hemoglobin A1c
CDEs: certified diabetes educators
SV: MEQNET SMBG Viewer (an originally developed data management software)
MARD: mean absolute relative difference
QOL: quality of life
